# Beyond the Parallel-Group Randomised Trial: A Portfolio of Designs for Cerebrovascular Neurosurgery

**DOI:** 10.1159/ned/adwag029

**Published:** 2026-09-09

**Authors:** Heze Han, Alejandro A. Rabinstein, Shuo Wang, Xiaolin Chen, Victor Volovici

**Affiliations:** a Department of Neurosurgery, Beijing Tiantan Hospital, Capital Medical University, Beijing, China; b Department of Neurosurgery, Erasmus MC Stroke Center, Erasmus University Medical Center, Rotterdam, The Netherlands; c Center for Complex Microvascular Surgery, Erasmus University Medical Center, Rotterdam, The Netherlands; d Department of Neurology, Mayo Clinic, Rochester, MN, USA

**Keywords:** Randomised controlled trial, Trial design, Cerebrovascular disease, Neurosurgery, Adaptive trial

## Abstract

**Introduction:**

The randomised parallel-group trial is the dominant evaluation instrument in evidence-based medicine, yet several cerebrovascular diseases violate most of the assumptions on which it depends. Unruptured arteriovenous malformations, unruptured intracranial aneurysms, asymptomatic carotid stenosis, and symptomatic haemodynamic carotid or middle cerebral artery occlusion are rare; their natural histories extend across decades; their treatments concentrate substantial harm at a single moment; outcomes depend on operator skill and infrastructure; and the choice between operating and waiting often turns on patient values. The resulting evidence base is sparse, is inconsistent, and rarely changes practice.

**Methods:**

This evidentiary deficit cannot be resolved by performing more conventional trials but by widening the portfolio of designs. After characterising the structural features that make the parallel-group trial inadequate here, we describe five proposals: expertise-based cohort-embedded randomisation, plaque-progression-enriched randomisation, a Bayesian sequential three-arm trial, a stepped-wedge cluster trial, and a synthesising, aspirational multi-arm Bayesian platform that shares a continuously updated natural-history control across strata. For each proposal, we set out the eligibility logic, the analytic engine, and the operational obstacles and verify illustrative sample-size assumptions by Monte Carlo simulation where applicable.

**Conclusion:**

Cerebrovascular disease requires a wider design vocabulary than the parallel-group RCT. Expertise-based randomisation, cohort embedding, adaptive enrichment, Bayesian inference, stepped-wedge allocation, and platform trials are established tools that address rarity, operator heterogeneity, long latency, and patient preference directly. Deploying them in cerebrovascular neuroepidemiology is achievable but will require registry infrastructure, sustained multi-cycle funding, and governance arrangements that do not yet exist in most settings.

## Introduction

The standard two-arm randomised trial is calibrated for diseases that are common, treatments that are easily administered, and effects that emerge within months. Several cerebrovascular disorders in neurosurgery fail every one of these assumptions, and the resulting mismatch generates a small set of recurrent obstacles that any methodological proposal must address.

Most of these diseases are rare. Unruptured arteriovenous malformations (AVMs) occur at roughly 1 per 100,000 person-years [1], and symptomatic chronic internal carotid or middle cerebral artery occlusions are rarer still. Intracranial aneurysms are common in the population (≈3% prevalence) [2], but those of intermediate rupture risk – the subset that actually requires a treatment decision – form a much smaller pool. The ARUBA trial illustrates the consequences: 226 of 1,740 screened patients enrolled across 39 sites in 9 countries [3]. The addressable population becomes thin very quickly once eligibility criteria are imposed.

Harm distributes very differently between the two arms in these conditions. An operation imposes a defined hazard within days, sometimes within hours; conservative management imposes a smaller annual hazard that does not terminate. For an unruptured AVM, annual haemorrhage risk is typically cited at 1–3% and accumulates over a patient’s remaining life [4]. For an unruptured aneurysm, 5-year rupture risk ranges from 0.4% in the lowest PHASES band to 17.8% in the highest, depending on size, location, and patient factors [5]. For an asymptomatic carotid stenosis, the relevant hazard is a low but persistent annual stroke risk that intensive medical therapy reduces but does not abolish [6]. Hazard ratios over a short follow-up window are therefore poor summaries of the choice the patient actually faces.

The treatment effect can also express itself slowly. CMOSS, which compared extracranial-intracranial bypass to medical therapy in patients with symptomatic chronic arterial occlusion, was neutral at its prespecified 2-year endpoint (8.6% versus 12.3%) and showed separation only at extended follow-up (11.2% versus 19.6% at a median of 7.6 years) [7, 8]. A correctly answered short-term question can leave the relevant clinical question untouched.

Surgical performance is not interchangeable across operators. A clinician who performs 80 aneurysm clippings annually but no coilings cannot represent both arms of a clipping-versus-coiling comparison. Trials such as ISAT addressed this by recruiting centres able to deliver both modalities [9], but the strategy breaks down whenever the expertise requirements differ across arms. And when the choice is between an acute, large procedural risk and an indefinite small annual one, patient values matter. Younger patients with low-grade lesions may reasonably prefer excision even at the cost of some complications; older patients with comparable lesions may prefer surveillance. Designs that ignore preference invite refusal at recruitment or crossover after randomisation – both of which occurred in ARUBA [3, 10].

Taken together, these features make the conventional parallel-group RCT the wrong default instrument for these diseases (Table 1). The methodological response is not to abandon randomisation but to use it differently. The Martin et al. [11] systematic review of 82 neurosurgical RCTs comparing surgery to non-operative management found that high methodological quality was inversely associated with demonstration of surgical benefit, a result that we read not as evidence against surgery but as evidence against the design conventions we have inherited.

**Table 1.** Disease-specific features relevant to trial design

Disease	Incidence/prevalence	Hazard structure	Decision drivers	Horizon
Unruptured AVM	≈1 per 100,000 person-years	1–3% annual risk haemorrhage; lifelong	Spetzler-Martin class, age, eloquence, patient preference	Decades
Asymptomatic carotid stenosis	≈1–2% of adults (≥50% stenosis); rises with age and vascular risk factors	≈0.5–1% annual ipsilateral stroke on intensive medical therapy; plaque-dependent	Stenosis progression, vessel-wall MRI plaque features, age, comorbidity	5–10 years
Unruptured intracranial aneurysm	≈3% adult prevalence; small subset at intermediate risk	5-Year rupture risk 1–3% (PHASES 4–8)	Size, location, prior SAH, smoking, hypertension, age	Decades
Symptomatic atherosclerotic ICA/MCA occlusion with haemodynamic insufficiency	Rare; <1 per 100,000 person-years for the trial-relevant subgroup	≈10–15% recurrent stroke at 2 years on medical therapy	PET/MR perfusion, prior infarct burden, age	5–10 years

AVM, arteriovenous malformation; ICA, internal carotid artery; MCA, middle cerebral artery; MR, magnetic resonance; PET, positron emission tomography; PHASES, Population, Hypertension, Age, Size of aneurysm, Earlier SAH, Site of aneurysm; SAH, subarachnoid haemorrhage.

Several design adaptations developed outside neurosurgery may directly address the obstacles presented above (Table 2). Expertise-based randomisation requires the operator delivering each patient to meet an expertise threshold for that arm [12], dissolving the within-surgeon equipoise problem while keeping the comparison treatment vs. treatment. The analogous restriction applies at the centre level only when the arms are infrastructurally exclusive (e.g., presence vs. absence of an extracranial-intracranial bypass programme). The IDEAL framework structures evaluation of innovative procedures so that centres reach randomisation only after an exploration stage with defined volume and outcome thresholds [13]. The cohort multiple randomised controlled trial design embeds randomisation in a prospective registry [14], allowing eligible cohort members to be offered the experimental intervention while the remainder continues as observational comparator. Stepped-wedge cluster designs randomise the time at which centres adopt an intervention, ensuring that all participating sites eventually deliver the experimental therapy [15]. Bayesian adaptive designs use prior distributions – derived, in our setting, from prospective registry estimates – to allow allocation to evolve as data accrue and offer formal mechanisms to drop arms that are unlikely to outperform alternatives [16]. Platform trials assemble several arms under a single master protocol, sharing one control and pooling administrative infrastructure [17].

**Table 2.** Design tools and the obstacles they address

Design tool	Obstacle it addresses	Design
Expertise-based randomisation	Within-surgeon equipoise; operator quality variance	Design 1 (AVM)
Cohort multiple randomised trial	Recruitment dilution; refusal; no contemporaneous natural-history comparator	Design 1; design 5
Adaptive enrichment with biomarker/progression threshold	Heterogeneity of natural history; ethical exposure of low-risk patients	Design 2 (carotid)
Bayesian sequential trial with informative prior	Insufficient sample size for multi-arm comparison; ethical drift toward better-performing arms	Design 3 (aneurysm)
Stepped-wedge cluster trial	Within-centre equipoise; long-horizon outcomes; ethical exposure to experimental therapy	Design 4 (EC-IC bypass)
Multi-arm Bayesian platform trial	All of the above; obsolescence of frozen single-trial protocols	Design 5 (platform)

AVM, arteriovenous malformation; EC-IC, extracranial-intracranial.

Each of these designs has an established role in oncology, critical care, or pragmatic chronic disease research. None has been seriously deployed in cerebrovascular surgery or even cerebrovascular disease. The remainder of this paper sets out five disease-specific applications. The first four address particular clinical questions; the fifth combines them into a single platform, for which we found no direct counterpart in the cerebrovascular literature.

## Methods

We propose five designs, each tailored to a specific disease and decision problem; their key features are summarised in Figure 1. The full statistical specification of each design is given in the online supplementary material (for all online suppl. material, see https://doi.org/10.1159/ned/adwag029).

**Fig. 1.**
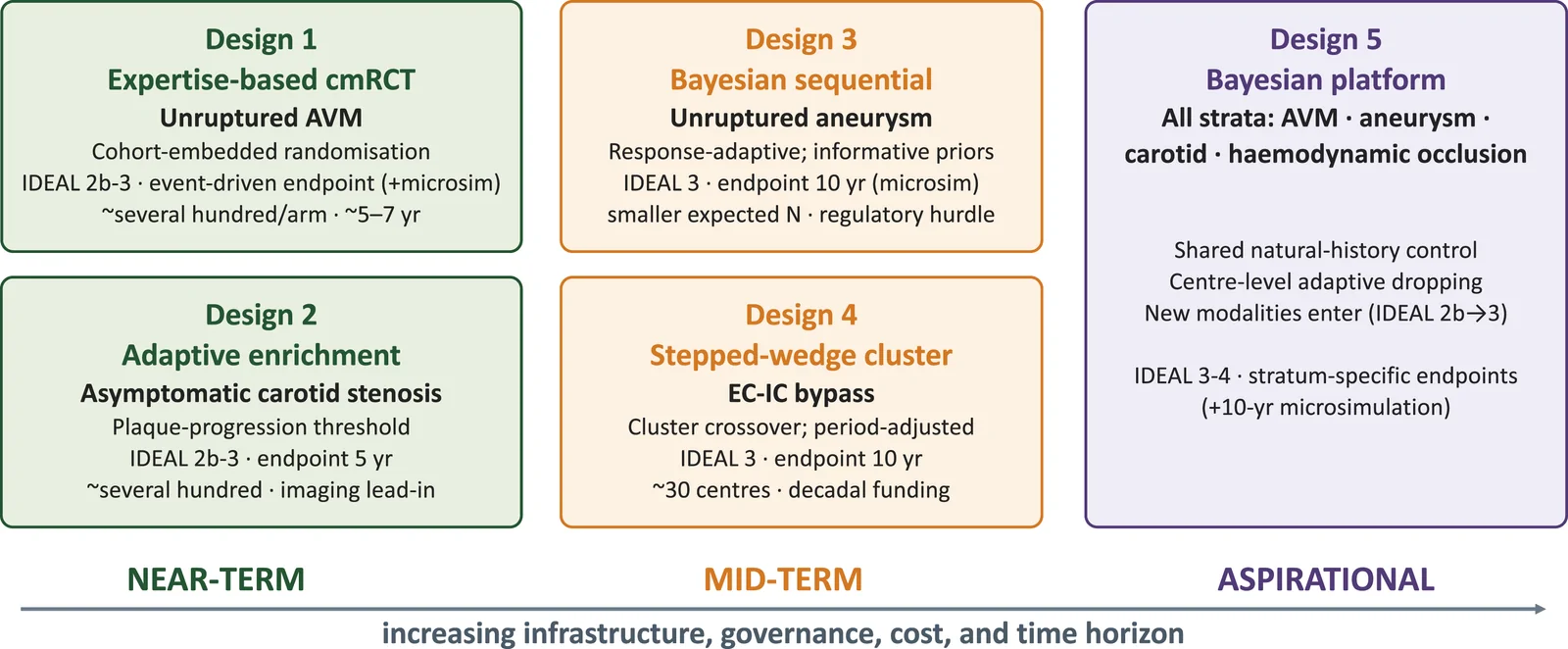
Staged roadmap of the five proposed designs.

### Design 1: Expertise-Based, Cohort-Embedded Randomisation for Unruptured AVMs

#### Clinical Question

In an adult with an unruptured AVM, does offered intervention by the modality appropriate to the Spetzler-Martin class – microsurgical resection for grade I, II, and some of III, multimodal staged treatment for the rest of III and IV, V – at a centre declared competent in that modality improve lifetime quality-adjusted survival relative to structured surveillance?

#### Why the Standard RCT Fails

ARUBA pooled different modalities within the interventional arm, allowed centres to select their own treatment without volume thresholds, and used a composite primary endpoint indifferent to the timing of harm [3, 10]. The result was an interventional arm that did not represent the best contemporary care, against a comparator in which hazard was not given enough time to accrue.

#### Proposed Design

Establish a prospective registry of all patients newly diagnosed with an unruptured AVM at participating centres. Centres declare modality competence with explicit annual volume thresholds, audited externally. Patients consenting to cohort participation are eligible, within the cohort multiple randomised controlled trial framework [14], to be offered randomisation. Randomisation is 1:1 between offered intervention and structured surveillance. Patients allocated to intervention are referred to a centre declared competent in the modality appropriate to their Spetzler-Martin grade. Patients allocated to surveillance receive a defined imaging and clinical follow-up schedule. The primary endpoint is the 5-year composite of stroke, death, or new persistent neurological deficit, with a mandatory microsimulation extrapolation to 20 years using class-specific haemorrhage rates derived from the same cohort.

#### Operational Notes

Sample size is event driven. Assuming ∼88% 5-year event-free survival under structured surveillance and a clinically meaningful improvement under offered grade-appropriate intervention [18], a two-sided log-rank test (α = 0.05, 80% power, 1:1) requires on the order of several hundred participants per arm, assuming ∼3 years of accrual and ∼6 years total duration (3–6 years of follow-up rather than a fixed 5-year window) with modest crossover and loss to follow-up; the exact figure is sensitive to the assumed effect and would be finalised against registry data. Power under this accrual was confirmed by Monte Carlo simulation across a grid of plausible effects, with crossover, front-loaded peri-procedural harm and loss to follow-up (online suppl. Fig. S1). We assume embedding within an existing prospective multicentre AVM registry network, such as MATCH or MARS, rather than de novo recruitment, and make this reliance explicit: following the reconstructed-cohort logic of Logroscino et al. [19], the network’s defined geographic catchment and referral system supply the person-time denominator and the natural-history incidence that anchor the 20-year microsimulation extension (online suppl. Material S1).

#### Pros

The design simultaneously addresses several criticisms to the ARUBA trial: treatment modality heterogeneity, variable operator quality, and narrow eligibility. The registry doubles as the natural-history estimator for the microsimulation. The trial uses real clinical referral logic, which preserves external validity.

#### Cons

Spetzler-Martin-grade-conditional referral within the intervention arm is not randomised, so the design compares “offered grade-appropriate intervention” against surveillance, not modality against modality. Registry infrastructure is expensive and slow to build.

### Design 2: Plaque-Progression-Enriched Randomisation for Asymptomatic Carotid Stenosis

#### Clinical Question

In an adult with asymptomatic atherosclerotic internal carotid artery stenosis, does revascularisation added to intensive medical therapy reduce 5-year ipsilateral ischaemic stroke compared with intensive medical therapy alone, when randomisation is restricted to patients whose plaque demonstrates progression or high-risk features on serial imaging?

#### Why the Standard RCT Fails

Most asymptomatic carotid stenoses never cause stroke under contemporary intensive medical therapy, and the annual ipsilateral stroke rate has fallen to roughly 0.5–1% [20]. Randomising every patient at the moment of detection dilutes any treatment effect toward zero and exposes a large, biologically quiescent majority to procedural stroke and cardiac risk. A trial that randomises on the degree of stenosis alone cannot answer a question whose answer depends on plaque biology and its trajectory over time.

#### Proposed Design

The proposed design is a two-stage adaptive enrichment trial. Stage 1 is a prospective observational cohort with standardised carotid imaging read centrally at baseline, 6 and 12 months, and annually thereafter. Patients whose plaque crosses a prespecified progression threshold become eligible for stage 2 randomisation between revascularisation plus intensive medical therapy and intensive medical therapy alone. Patients below the threshold remain in the observational cohort and contribute to the natural-history dataset that informs eligibility refinement.

#### Operational Notes

Assuming that ∼25% of a broad cohort crosses the progression threshold [21], and taking a 5-year ipsilateral stroke rate of ∼12% in the enriched medical-therapy stratum against a clinically meaningful reduction with revascularisation, the enriched randomisation requires on the order of several hundred participants drawn from a broad observational cohort of a few thousand. Central adjudication of the imaging biomarker and of the stroke endpoint is essential, as is a standardised, auditable definition of intensive medical therapy that is updated as the standard of care evolves (online suppl. Material S2).

#### Pros

Randomisation takes place when the biological indication for a decision actually emerges, sparing quiescent patients procedural harm. The observational stage generates natural-history evidence as a by-product. The medical-therapy-alone arm in stage 2 is ethically defensible precisely because plaque activity has been objectively documented, and the design nests alongside broad-eligibility trials.

#### Cons

The progression threshold is contested and may be relitigated mid-trial as vessel-wall imaging evolves. Vessel-wall MRI is not yet uniformly available and central reading is logistically demanding. Patients with documented plaque progression may decline continued medical therapy regardless of allocation.

### Design 3: Bayesian Sequential Three-Arm Trial for Treatment Selection in Unruptured Aneurysms

#### Clinical Question

In a patient with an unruptured intracranial aneurysm of intermediate rupture risk (operationally, a PHASES score of 4–8, corresponding to a 5-year rupture risk of approximately 1–3%, with reasonable life expectancy), is microsurgical clipping, endovascular treatment, or structured surveillance the optimal initial strategy when measured over a 10-year horizon?

#### Why the Standard RCT Fails

ISAT addressed a different decision by randomising patients with ruptured aneurysms [9]. ISUIA was observational [22]. A frequentist three-arm trial powered for the conventional level requires impractically high numbers, particularly when long-term outcomes are the primary interest. Sequential trial conduct also exposes the field to ethical objection if early results clearly favour one arm.

#### Proposed Design

The proposed design is a Bayesian sequential trial with informative priors. Priors for arm-specific rupture, periprocedural, and long-term disability rates are constructed from published natural-history evidence – including ISUIA [22] and PHASES [5] cohorts – and from contemporaneous procedural registries. Patients are randomised 1:1:1 to clipping, endovascular treatment, or structured surveillance. The trial proceeds in blocks; after each block (for instance, every 60 patients), the posterior probability that each arm is the optimal long-term strategy is updated. Allocation in the next block is response adaptive, weighted towards arms with higher posterior probability of optimality. Arms whose posterior probability of being optimal falls below a prespecified stopping threshold are dropped. The primary endpoint is 10-year disability-free survival, projected for participants with shorter follow-up via microsimulation calibrated to the trial’s own observed early outcomes.

#### Operational Notes

The total sample size is variable by construction; the design’s principal statistical advantage is that the expected sample size to reach a valid conclusion is substantially smaller than the frequentist three-arm equivalent. Pre-registration of the prior, the stopping rule, and the simulation engine are essential (online suppl. Material S3; Table S1).

#### Pros

The inferential machinery makes explicit use of the same natural-history evidence on which the clinical decision already relies. Expected sample-size reduction is a potential advantage. There is ethical attractiveness in moving patients towards better-performing arms as evidence accrues.

#### Cons

Some regulators and editors remain wary of Bayesian inference. The prior is open to legitimate dispute. Response-adaptive allocation introduces time-varying confounding if secular trends in medical therapy or in procedural technique are large. Centres with low aneurysm volume may receive few allocations and be effectively dropped from the trial without a formal mechanism.

### Design 4: Stepped-Wedge Cluster Trial for Extracranial-Intracranial Bypass

#### Clinical Question

In symptomatic atherosclerotic carotid or middle cerebral artery occlusion with PET- or MR-perfusion-confirmed haemodynamic insufficiency, does the introduction of extracranial-intracranial bypass into a centre’s care pathway reduce 7-year ipsilateral ischaemic stroke incidence?

#### Why the Standard RCT Fails

COSS [23] and CMOSS [7] were both individually randomised parallel-group trials. Both struggled with eligibility narrowness; both fixed an outcome window that captured procedural harm but not the long-term benefit signal that subsequently emerged [8]. Individual-patient randomisation in this small population also collides with strong physician preferences.

#### Proposed Design

The proposed design is a stepped-wedge cluster randomised trial. Centres meeting expertise and volume thresholds enter in a “medical therapy-only” period. They cross over to a “bypass-eligible” period at randomised time points, until all centres are in the bypass-eligible period. All eligible patients within a centre during a given period receive that period’s standard of care. The primary endpoint is time to ipsilateral ischaemic stroke, analysed with adjustment for time period to control secular trends in medical therapy, with a common administrative close at 10 years from first randomisation.

#### Operational Notes

Assume 30 centres meeting expertise and volume thresholds, each contributing 10 eligible patients per year, randomised to 5 sequences of 6 centres. The trial runs over 6 periods of 10 months: all centres are medical only in the first period, and one sequence crosses over at each of the five subsequent steps, so that 90 of the 180 cluster periods are bypass-eligible. This gives approximately 8 patients per cluster period and about 1,500 randomised patients accrued over a 5-year wedge. With a common administrative close at 10 years, follow-up ranges from 5 to 10 years (mean 7.5) and yields approximately 255 primary events. Centres commit prospectively to deliver bypass at a defined standard once they cross over (online suppl. Material S4).

#### Pros

Pros are as follows:

- avoids the within-centre equipoise problem;
- every participating centre eventually delivers the experimental therapy, which materially improves recruitment and retention;
- captures the long-term benefit signal that two prior trials missed;
- reflects how interventions are actually introduced into practice.

#### Cons

Centre-level confounding by case mix is difficult to eliminate without large numbers of clusters. Funding cycles rarely match a 10-year horizon. Clinicians at crossed-over centres may also be reluctant to offer bypass to borderline-eligible patients. Mitigation requires pre-crossover training, public reporting of per-centre intervention rates among eligible patients, and protocol-level definitions of “borderline eligibility” that constrain individual discretion.

### Design 5: A Multi-Arm Bayesian Platform Trial across Cerebrovascular Diseases

The four proposals above each address a single disease in isolation. None makes use of the fact that the eligible populations overlap in catchment, share infrastructure, and depend on overlapping prognostic models. We now describe a novel synthesising design in the field of cerebrovascular surgery. The first four designs above are methodologically established outside cerebrovascular research but unused within it; the fifth is additionally methodologically novel in its centre-level adaptive mechanism.

#### Structure

A single-master protocol covers four disease strata: unruptured AVM, unruptured aneurysm, asymptomatic carotid stenosis, and symptomatic carotid/MCA occlusion with haemodynamic insufficiency. Within each stratum, patients are eligible for randomisation among the modalities applicable to their condition (microsurgery, endovascular, radiosurgery, bypass, surveillance, medical therapy alone) at centres that have declared expertise in those modalities, with pre-specified per-trial volume and outcome benchmarks consistent with IDEAL stage 3 criteria [24].

#### Shared Natural-History Control

All four strata draw their conservative-management comparator from a continuously updated prospective registry embedded in the same participating centres. Patients who decline randomisation, or who fall outside randomisation eligibility, enter the registry under the same data collection schedule. The registry’s hazard estimates are used as Bayesian priors for the corresponding stratum’s randomised analysis. Because the registry continues to accrue indefinitely, the priors strengthen over time, addressing the small-sample precision problem that has defined the field.

#### Centre-Level Response-Adaptive Allocation

Within each stratum, each participating centre’s recent performance on each modality is summarised by a posterior probability of optimal outcome conditional on patient class. Allocation is adjusted not for individual patients but for centres: when a centre’s posterior probability of optimal performance in a given modality falls below a prespecified threshold, that modality is dropped from that centre’s offering. The trial’s headline comparisons therefore stay clean, while underperforming combinations are removed where they are actually occurring. Patient-level response-adaptive allocation, which conflates treatment effect with secular trend, is deliberately avoided.

#### Admission of New Modalities

New modalities such as transvenous AVM embolisation, intrasaccular aneurysm devices, and new flow-diverter generations enter the platform once they have completed IDEAL stage 2b exploration and meet the readiness criteria for stage 3 randomised assessment [24]. Each new arm begins with a small dedicated allocation and grows or contracts under the same response-adaptive rules. The platform therefore evolves with the field rather than freezing a protocol decade.

#### Outcomes

Stratum-specific primary endpoints are each required to include a 10-year microsimulation extension. Patient-reported outcomes (functional status, anxiety, employment) are common across strata to enable cross-stratum modelling of how patients value the trade-off between concentrated and distributed risk.

#### Implementation

We see implementation in three phases. Phase 1: establish the shared registry across 10–20 high-volume centres for 2 years, agreeing on data definitions, imaging acquisition, and outcome adjudication. Phase 2: open randomisation in one stratum – we suggest unruptured AVM because the existing trials are most contested and the equipoise is most genuine – and test the centre-level adaptive machinery on that stratum alone. Phase 3: open the remaining strata sequentially over the following 3 years. Governance requires a single international coordinating committee, with stratum-specific scientific committees and a data and safety monitoring board with explicit authority to enforce centre-level modality drops (online suppl. Material S5).

#### Pros

Pros are as follows:

- addresses the small-sample problem by sharing controls across strata and over time;
- addresses the heterogeneous-intervention problem by tying modality to centre expertise and dropping under-performers;
- addresses the slow-effect problem by mandating extrapolation;
- evolves with the field rather than against it;
- provides natural-history estimates as a continuous public good.

#### Cons

Governance will be challenging and onerous. The statistical analysis plan must be specified in unusual detail and pre-registered. Regulators have limited precedent for cerebrovascular platform trials. Centre-level adaptive dropping introduces ethical and political tensions since modalities also carry revenue and institutional identity. International coordination across reimbursement systems is operationally demanding. We do not pretend any of this is easy. We argue, however, that it is more tractable than continuing to produce single trials whose conclusions are contested before the ink dries.

### Limitations

The five proposals are not equally ready for deployment (Fig. 1). Designs 1 and 2 are near term: both rest on registry and imaging infrastructure that already exists in several networks, and both could open within 2 to 3 years of funding, reporting primary results at approximately 6 and 7 years, respectively. Designs 3 and 4 are mid-term: the Bayesian sequential trial requires prospective agreement on priors and regulatory engagement before it can begin, and the stepped-wedge bypass trial requires a funding commitment spanning roughly a decade across some 30 centres, so realistic reporting horizons are 10 years or more. Design 5 is aspirational in the strict sense: its first phase of the shared registry is achievable now, but the full platform presupposes governance arrangements, data-sharing agreements, and analytic capacity that would take a decade to assemble even under favourable conditions. And the designs above share a small number of structural vulnerabilities.

#### Centre-Level Confounding

Because allocation to a modality is tied to centre expertise in the AVM cohort-embedded trial and in the platform, differences in case-mix and peri-procedural quality between centres can masquerade as treatment effects. This is mitigated by externally audited volume and outcome thresholds, by stratification and adjustment for centre, and – in the platform – by confining headline comparisons to within-centre contrasts and using the shared registry to model centre effects explicitly.

#### Threshold Misclassification

The carotid enrichment design randomises on a biomarker (plaque progression, intraplaque haemorrhage) measured with error; misclassification near the threshold both dilutes the enriched effect and distorts eligibility. Central adjudicated reading, pre-specified measurement error and sensitivity analyses around the cut point, and a lead-in cohort that recalibrates the threshold against observed events are the principal safeguards.

#### Time-Varying Confounding

When allocation adapts as data accrue, as in the Bayesian sequential aneurysm trial, secular change in procedural technique or medical therapy becomes entangled with the treatment effect. We adjust for calendar time, avoid patient-level response-adaptive allocation where it would conflate effect with trend, and pre-register the adaptation rule and priors.

#### Contamination and Secular Trend

In the stepped-wedge bypass design the experimental therapy may diffuse into control periods before crossover, while the background standard of care improves over a long horizon; both erode the contrast, and the same secular threat applies to the carotid design. Time-adjusted analysis, public per-centre intervention-rate reporting, protocol definitions of borderline eligibility, and pre-crossover training constrain these effects.

#### Extrapolation Dependence

Every design that carries a microsimulation extension rests, for its long-horizon conclusions, on modelled hazards beyond the observed follow-up. We calibrate the microsimulation to each trial’s own early outcomes and report its assumptions and sensitivity explicitly rather than as a single projected curve.

#### Registry Selection

In the cohort-embedded and platform designs, patients selecting into or out of randomisation for unobserved reasons bias the natural-history comparator. Inverse-probability weighting, capture of the full registry denominator in the reconstructed-cohort sense [19], and comparison of randomised and registry-only strata on measured covariates are required corrections.

None of these threats is unique to the designs we propose; each has an established remedy in the methodological literature. Making them explicit, and matching each remedy to the design that needs it, is itself part of moving from conventional trials towards designs fitted to the disease.

## Discussion

Researchers in cerebrovascular disease have produced individually sophisticated trials. Yet they have not produced the design diversity required by some of the diseases they treat. Each landmark trial in our field answered a particular question competently, and each was promptly extended in clinical practice to questions it did not address. The result is an evidence base whose authority is contested at every meeting and whose adoption in practice is only modest [11].

The remedy is not larger trials of the same form. It is designing different kinds of trials. The methodological literature has already supplied them in other fields. Their limited uptake in cerebrovascular and neurosurgical research is not, in our view, explained by any property of the methods themselves. It reflects a combination of structural factors: the small number of trial methodologists embedded in surgical research groups, the absence of the registry infrastructure on which cohort-embedded and adaptive designs depend, and funding cycles poorly matched to decade-long outcome horizons. None of these is a criticism of individual investigators, and all of them are addressable.

Neuroepidemiology is the natural setting in which to develop these designs. The longitudinal registries, the population-based vantage, and the multi-centre infrastructure required are already its working materials. We invite the community to consider the proposals here as a starting framework rather than a finished blueprint, to challenge their specifics, and — most importantly — to attempt them. The cost of continuing the present course is borne by patients, who continue to receive whichever treatment option their referral path provides, and by clinicians, who must improvise care with the lingering uncertainty of contested trials. The cost of changing course is the need for rigorous governance, sustained collaboration, and greater statistical ambition. The first cost is enduring; the second is finite.

## Statement of Ethics

This is a methodological article that does not involve human participants, human data, or human tissue. Ethics Committee approval and informed consent were therefore not required.

## Conflict of Interest Statement

V.V. serves as a member of Editorial Board for Neuroepidemiology; he was not involved in the editorial handling or peer review of this manuscript.

## Funding Sources

This study was not supported by any sponsor or funder.

## Author Contributions

H.H.: conceptualisation, methodology, simulation design and implementation, writing – original draft, and writing – review and editing. A.A.R.: conceptualisation, methodology, and writing – review and editing. S.W. and X.C.: methodology and writing – review and editing. V.V.: conceptualisation, methodology, supervision, writing – original draft, and writing – review and editing. All authors approved the final version of the manuscript.

## Data Availability

No primary patient-level data were generated or analysed in this methodological article.
